# Parapatric speciation of *Meiothermus* in serpentinite-hosted aquifers in Oman

**DOI:** 10.3389/fmicb.2023.1138656

**Published:** 2023-04-12

**Authors:** Mason Munro-Ehrlich, Daniel B. Nothaft, Elizabeth M. Fones, Juerg M. Matter, Alexis S. Templeton, Eric S. Boyd

**Affiliations:** ^1^Department of Microbiology and Cell Biology, Montana State University, Bozeman, MT, United States; ^2^Department of Geosciences, University of Colorado, Boulder, Boulder, CO, United States; ^3^School of Ocean and Earth Science, University of Southampton, Southampton, United Kingdom

**Keywords:** subsurface, serpentinite, recombination, evolution, geographic isolation, parapatric speciation, dispersal limitation, competitive exclusion

## Abstract

The factors that control the distribution and evolution of microbial life in subsurface environments remain enigmatic due to challenges associated with sampling fluids from discrete depth intervals via boreholes while avoiding mixing of fluids. Here, using an inflatable packer system, fracture waters were isolated and collected from three discrete depth intervals spanning >130 m in a borehole intersecting an ultramafic rock formation undergoing serpentinization in the Samail Ophiolite, Sultanate of Oman. Near surface aquifer waters were moderately reducing and had alkaline pH while deeper aquifer waters were reduced and had hyperalkaline pH, indicating extensive influence by serpentinization. Metagenomic sequencing and analysis of DNA from filtered biomass collected from discrete depth intervals revealed an abundance of aerobes in near surface waters and a greater proportion of anaerobes at depth. Yet the abundance of the putatively obligate aerobe, *Meiothermus*, increased with depth, providing an opportunity to evaluate the influence of chemical and spatial variation on its distribution and speciation. Two clades of *Meiothermus* metagenome assembled genomes (MAGs) were identified that correspond to surface and deep populations termed Types I (S) and II (D), respectively; both clades comprised an apparently Oman-specific lineage indicating a common ancestor. Type II (D) clade MAGs encoded fewer genes and were undergoing slower genome replication as inferred from read mapping. Further, single nucleotide variants (SNVs) and mobile genetic elements identified among MAGs revealed detectable, albeit limited, evidence for gene flow/recombination between spatially segregated Type I (S) and Type II (D) populations. Together, these observations indicate that chemical variation generated by serpentinization, combined with physical barriers that reduce/limit dispersal and gene flow, allowed for the parapatric speciation of *Meiothermus* in the Samail Ophiolite or a geologic precursor. Further, *Meiothermus* genomic data suggest that deep and shallow aquifer fluids in the Samail Ophiolite may mix over shorter time scales than has been previously estimated from geochemical data.

## Introduction

The terrestrial subsurface is host to an abundant and active microbial biosphere (McMahon and Parnell, 2014; Bar-On et al., 2018; Magnabosco et al., 2018) that comprises populations of cells inhabiting the pore spaces and (micro)fractures of rocks (Goordial et al., 2021; Fones et al., 2022). A large amount of the habitable subsurface is comprised of mafic and ultramafic igneous rocks. Mafic and ultramafic igneous rocks can undergo the geologic process of serpentinization that can generate H_2_ through the oxidation of Fe(II)-containing minerals, such as olivine and brucite (Miller et al., 2017; Ellison et al., 2021). H_2_, in turn, can react with CO_2_ to generate formate (HCOO^–^), carbon monoxide (CO), or methane (CH_4_) (Seewald et al., 2006; McCollom and Seewald, 2007) that can be used as electron donors and/or carbon sources in microbial metabolism.

Serpentinization also generates highly reducing conditions that can limit the availability of oxidants needed to support microbial metabolism. For example, fracture fluids from rock formations undergoing serpentinization can exhibit oxidation-reduction potentials (*Eh*) as low as −750 mV (Templeton et al., 2021). Available oxidants in waters infiltrating these environments are consumed either abiotically via reactions involving minerals or soluble reductants (e.g., ferrous iron or hydrogen) or biotically by microorganisms. Low oxygen fugacity and oxidant limitation is characteristic of fluids collected from environments undergoing serpentinization (Frost and Beard, 2007; Rempfert et al., 2017; Suzuki et al., 2017; Templeton et al., 2021). In these systems, dissolved O_2_ concentrations rarely exceed 40 μM even in the least reacted waters and are below detection in highly reacted waters (Rempfert et al., 2017; Suzuki et al., 2017; Piccoli et al., 2019). Yet, despite the apparent absence of O_2_ in reacted waters, putatively obligate aerobes are often detected. For example, sequences related to the obligately aerobic genus of bacteria, *Meiothermus* (Deinococcota), have been detected in subsurface hyperalkaline and suboxic environments, oftentimes in high relative abundance (Chung et al., 1997; López-López et al., 2015; Woycheese et al., 2015). Likewise, fracture waters collected from a variety of depths in numerous wells have been shown to host *Meiothermus* DNA, including circumneutral near-surface waters and the most hyperalkaline and most highly reacted waters in the Samail Ophiolite, Sultanate of Oman (Rempfert et al., 2017; Nothaft et al., 2021).

Multiple reports of the presence of putative aerobes (e.g., *Meiothermus*) in what appear to be otherwise anoxic serpentinite fluids suggest the possibility that aerobes are widespread, can freely disperse among fractures in rocks, and are inactive in reduced portions of the serpentinites only to become active when favorable conditions are encountered. Depending on the time scales over which this occurs, the extent of dispersal could either allow for or fully restrict gene flow/recombination among otherwise isolated populations, allowing for localized speciation among closely related populations. Several recent studies offer differing perspectives on whether dispersal and/or gene flow/recombination are possible in subsurface habitats. A metagenomic analysis of low biomass subseafloor sediment communities in North Pond in the Atlantic Ocean sampled repeatedly (*n* = 10) over a period of nearly two years showed strain level shifts in the composition of populations comprising those communities (Anderson et al., 2022). Similarly, an analysis of 16S rRNA genes recovered from fluids in three boreholes intersecting a 1,400 m deep hard rock aquifer sampled over a 10 month period revealed substantial changes in community compositions (Zhang et al., 2022). In both studies, the results were interpreted to reflect dispersal of cells through sediment porewaters or fracture networks. In contrast, a recent study of 16S rRNA genes collected from subsurface fluids in the Cedars, a hard rock serpentinite formation located in California, USA, over multiple years concluded that spatial barriers limit dispersal (Putman et al., 2021). Together, these observations raise questions as to the predominant mode of speciation that drives microbial evolution in serpentinite formations, as well as other hard rock subsurface environments.

In 2017, the Oman Drilling Project drilled six new wells to establish a multi-borehole observatory of the serpentinizing subsurface in the Samail Ophiolite. In 2018 and again in 2019, a submersible down borehole packer and pump system was installed in several of the boreholes, including BA1A, a 400 m borehole that primarily intersects dunite nearer to the surface and harzburgite at depth (Lods et al., 2020; Malvoisin et al., 2020). The packer system uses inflatable nitrogen balloons to seal off permeable zones in the bedrock and to identify the primary fractures that allow for fluid flow. In BA1A, these were identified as 0–30 m, 41–65 m, and 108–132 m, with limited connectivity to aquifers below this depth (Lods et al., 2020). By sealing off these discrete intervals, fluids could be sampled while limiting the possibility for mixing of subsurface waters during sampling within the well. Previously reported chemical analyses reveal aquifer waters from the top two depth intervals in BA1A are circumneutral and moderately reducing whereas aquifer waters from the deeper interval are hyperalkaline and highly reducing, consistent with successful segregation of fluid types by the packer system (Nothaft et al., 2021). Furthermore, 16S rRNA gene sequencing of biomass collected from each of the three discrete depth intervals revealed shifts in the composition of the communities from largely aerobic taxa nearer to the surface toward anaerobic, putatively sulfate reducing taxa at depth (Nothaft et al., 2021). Notably, *Meiothermus* sequences were detected at all three sampled depths, but had an order of magnitude higher relative abundance in the 108–132 m interval. This suggests that the presence of *Meiothermus* is unlikely to be due solely to mixing of fluids during sampling and raises the question of how *Meiothermus* withstand the polyextremophilic conditions associated with heavily serpentinized deep subsurface fluids, including an apparent lack of O_2_.

Here, we apply metagenomic sequencing to DNA extracted from biomass from each of the three isolated depth intervals (0–30 m, 41–65 m, and 108–132 m) in BA1A to further evaluate controls on the distribution and evolution of microbial life in subsurface environments undergoing serpentinization, with a specific focus on *Meiothermus*. Sequences were assembled, binned into metagenome assembled genomes (MAGs), and *Meiothermus*-affiliated MAGs were identified and curated. *Meiothermus* MAGs were then compared bioinformatically, phylogenetically, and metabolically to identify similarities and differences as a function of depth and extent of serpentinization in the fluids they inhabited. Estimated genome replication rates were calculated for *Meiothermus* MAGs to establish whether those populations are likely in an active state of replication. Single nucleotide variants (SNVs) were identified to generate high fidelity *Meiothermus* population structures to evaluate patterns in the diversification and gene flow/recombination among depth-resolved populations. Collectively, the results indicate that chemical and physical barriers that limit co-habitation and gene flow/recombination, in combination with gene loss and gain via mobile genetic elements, drove parapatric speciation of *Meiothermus* in the Samail Ophiolite. While gene flow/recombination among depth-resolved populations was limited, it was detected, suggesting that the reduced, hyperalkaline subsurface aquifer waters in the Samail Ophiolite may not be as isolated from the surface aquifer as has been previously estimated based on bulk characterization of the chemistry of fluids [e.g., deep fluids isolated for >20,000 years (Paukert Vankeuren et al., 2019)]. The results are discussed in terms of processes controlling the distribution and evolution of microbial populations in subsurface environments undergoing serpentinization.

## Materials and methods

### Site description, drilling, and sampling

BA1A is a part of a multi-borehole observatory established by the Oman Drilling Project. Details on drilling are reported previously (Lods et al., 2020; Kelemen et al., 2021; Nothaft et al., 2021) and are further expanded upon in the Supplementary methods. Briefly, BA1A is a 400 m deep, 0.152 m diameter well, intersecting fully serpentinized dunite in the upper 250 m and partially serpentinized harzburgite at greater depth (Lods et al., 2020; Kelemen et al., 2021).

A Solexperts packer system (Zurich, Switzerland) that included two inflatable bladders (“packers”) and a Grundfos (Bjerringbro, Denmark) model SQE 1–140 submersible pump was installed in BA1A in February 2019, enabling the isolation of discrete depth intervals for hydrological testing (Lods et al., 2020) and microbiological and chemical characterization of groundwaters (Nothaft et al., 2021). Samples of planktonic biomass were collected from discrete depth intervals in February 2019 using the packer system, as described previously (Nothaft et al., 2021). Controls for contamination, both in the form of remnant drilling fluid as well as laboratory contamination and DNA extraction controls, are described in previous studies (Nothaft et al., 2021; Templeton et al., 2021).

### DNA extraction and shotgun metagenomic sequencing

Genomic DNA was extracted from filtered biomass with the Qiagen PowerSoil kit (Germantown, MD, USA) and was submitted to the University of Wisconsin Biotechnology Center for library preparation following the Illumina (San Diego, CA, USA) regular fragment (∼300 bp) kit and these libraries were shotgun sequenced via the Illumina NovaSeq 6000 (2 × 150 bp) platform. Information on the depth and quality of sequences obtained from the three libraries are reported in Supplementary Table 1. Additional details on the DNA extraction can be found in Nothaft et al. (2021) and in the Supplementary methods.

### Metagenomic sequence assembly, binning, and metagenome assembled genome (MAG) metabolic predictions

DNA was shotgun sequenced, curated, assembled, and binned into MAGs using the same pipeline outlined previously (Fernandes-Martins et al., 2021). Further details regarding assembly and binning can be found in the Supplementary methods. MAGs are available from National Center for Biotechnology Information (NCBI) under the BioProject identification number PRJNA918706. The taxonomic affiliations, relative abundances, and completeness/quality of MAGs in each of the three communities are reported in Supplementary Table 2.

Metagenome assembled genomes were characterized as corresponding to putatively aerobic or anaerobic cells using the Basic Local Alignment Search Tool (BLASTp) to query MAGs first for homologs of cytochrome *c* oxidase [Cox I and II; Enzyme Category (EC) 7.1.1.9] and cytochrome *bd* complex (CydABX; EC 7.1.1.7), two proteins necessary for aerobic metabolism. Following this, MAGs were uploaded to the Kyoto Encyclopedia of Genes and Genomes (KEGG) server for annotation and pathway prediction. Specifically, orthologs predicted by KEGG to be involved in other (i.e., those not involving O_2_) aspects of putative electron transport chains were examined, specifically orthologs of terminal oxidases (e.g., dissimilatory nitrate reductase, dissimilatory bisulfite reductase). The potential for a MAG to correspond to an aerobe or an anaerobe was then cross checked against the metabolism of its closest cultivated relative, as assessed using the Genome Taxonomy Database-Toolkit (GTDB-Tk) (Chaumeil et al., 2019). Further details regarding this approach can be found in the Supplementary methods. We also evaluated the potential for alternative O_2_-producing biochemical mechanisms in BA1A populations. MAGs were examined for genes encoding enzymes known to produce O_2_, including nitric oxide dismutase (Nod; Zhu et al., 2019), superoxide dismutase (SOD; Imlay, 2002), chlorite dismutase (Cld; Hofbauer et al., 2014), and peroxidases/catalases (Cat; Singh et al., 2008) using BLASTp, with characterized proteins as query sequences (Supplementary Table 3).

### Compilation of *Meiothermus* genomes and MAGs

Metagenome assembled genomes were assigned taxonomy using the GTDB-Tk (Chaumeil et al., 2019). Using this approach, MAGs that showed close affiliation to *Meiothermus* were compiled from BA1A metagenomes, as well as fracture fluid metagenomes from other subsurface locations in the Samail Ophiolite (Fones et al., 2019, 2021). Taxonomic assignments were verified manually via BLASTp analysis of housekeeping genes (e.g., RNA polymerase) against the NCBI non-redundant database.

The size of *Meiothermus* MAGs was estimated by normalizing MAG size to estimated completeness, where completeness was determined as the proportion of housekeeping genes present as determined by Metawrap (version 1.3) (Uritskiy et al., 2018). The housekeeping genes assumed to be present in a complete genome were expected to be the same as those present in *Meiothermus hypogaeus* the closest relative (∼85% ANI) to Oman *Meiothermus* strains. Only MAGs that contained >60 full length housekeeping genes were retained for downstream analysis. This included six *Meiothermus* MAGs from BA1A and twelve *Meiothermus* MAGs from our database of Oman fracture fluid metagenomes (Fones et al., 2019, 2021).

### Phylogenomic analyses of *Meiothermus*-affiliated MAGs

The database of Oman *Meiothermus* MAGs was subjected to alignment of housekeeping genes using the GTDB-Tk (Chaumeil et al., 2019). Also included in the alignments were housekeeping genes from eight *Meiothermus* lineages not from Oman (*Meiothermus taiwanensis* GCA_000482765.1, *Meiothermus ruber* GCA_015478585.1, *Meiothermus silvanus* GCF_000092125.1, *Meiothermus rufus* GCF_00042325.1, *Meiothermus cerbereus* GCF_000620065.1, *Meiothermus* sp. Pnk-1 GCF_003226535.1, *Meiothermus* sp. QL-1 GCF_003351145.1, and *Meiothermus hypogaeus* GCF_003574035.1) to be used as an outgroup. The alignment was generated using Clustal Omega (version 1.2.4) (Sievers et al., 2011), and IQtree (version 1.6.12) (Minh et al., 2020) was used to generate the tree specifying the LG substitution model and 1,000 bootstraps. The tree was visualized using the Interactive Tree of Life (iTOL) web platform (version 6) (Letunic and Bork, 2021), which identified two clades of *Meiothermus* that are herein referred to as “Type I surface or (S)” and “Type II deep or (D).”

Additionally, since housekeeping genes could not be retrieved for many other *Meiothermus* MAGs or metagenomic assemblies, RpoB sequences from MAGs closely related to *Meiothermus* and from unbinned metagenomic sequence data from other environmental samples were retrieved (Supplementary Table 4), including those from the NCBI non-redundant (NR) database and the sequence read archive (SRA) database. RpoB from *Thermus thermophilus*, a member of the sister genus to *Meiothermus* (Henne et al., 2004), was used to root the phylogeny. The tree was generated and visualized as described above.

### Estimation of *Meiothermus* genome replication rates

The Strain level Metagenomic Estimation of Growth (SMEG) program (ver. 1.1.1) was used to infer genome replication rates for MAGs that showed closest affiliation to *Meiothermus* (Emiola et al., 2020). Mauve (ver. 2.4) (Darling et al., 2004) was used to reorder contigs of *Meiothermus*-affiliated MAGs through alignment to the genome of *M. hypogaeus*. The quality of contig realignment was examined by evaluating the depth of mapped raw reads to ensure that they were generally reordered from highest read depth (origin of replication) to lowest read depth (terminus of replication). SMEG then aligned these reordered contigs to determine single nucleotide variants (SNVs) and assigned MAGs into phylogenetic subclusters based on SNV sites. Read coverage for each contig within each MAG at each SNV site was then determined, and the resultant read map coverage ratios were used to estimate genome replication rates.

### Assignment and characterization of *Meiothermus* protein clusters

Protein clustering was used to identify protein encoding genes unique to each *Meiothermus* MAG and to facilitate downstream evaluation of SNV profiles. All called proteins for each MAG were subjected to protein clustering using CD-HIT (ver. 4.8.1) (Fu et al., 2012) resulting in clusters of homologous proteins. Additional details regarding the protein clustering approach used herein can be found in the Supplementary methods.

Representative sequences from each protein cluster among BA1A *Meiothermus* MAGs were uploaded to KEGG and analyzed using the BlastKOALA annotation pipeline (Kanehisa et al., 2020). This pipeline assigns hierarchical annotations to protein sequences, wherein each uploaded protein sequence is annotated with the closest database protein, and then is placed into a hierarchy of biological pathways. These annotations were collated and compared across the six *Meiothermus* MAGs from BA1A to identify which KEGG orthologs, protein families, and biological pathways were overrepresented in certain MAGs or MAG groupings. Differences between the Type I (S) and Type II (D) clades were investigated, as were differences between more abundant and less abundant (i.e., rare) MAGs.

### Identification and curation of single nucleotide variants (SNVs)

Single nucleotide variants were identified in the six high quality BA1A *Meiothermus* MAGs using Instrain (ver. 1.5.5) (Olm et al., 2021). Instrain identifies SNVs in metagenomic data by mapping the trimmed metagenomic reads against a provided reference genome. Since the BA1A *Meiothermus* MAGs are not closely related (<90% ANI) to any published reference genome, each of the six MAGs was used as a reference genome for SNV identification. SNVs were identified among the six MAGs, and these were then curated to identify and remove those that likely result from sequence read errors. Specifically, putative sequence errors were identified by calculating the ANI between sequence read pairs and the reference genome. Read pairs exhibiting pairwise ANI to the reference genome of <90% were discarded. This ensured that read pairs bearing a significant mismatch were not used for SNV calling, as this would likely result in the retrieval of false positive SNVs. The minimum consensus score was set to 90% rather than 95% to account for lower overall quality of metagenomic data relative to genomes from pure cultures.

Single nucleotide variants identified as a consensus SNV [i.e., SNVs where the variant base frequency was present in 40% of mapped reads or more (see below)] were also identified. Consensus SNVs were rare in the BA1A Oman *Meiothermus* MAGs and occurred at a frequency of about 1:25,000 base pairs (bp). Consensus SNVs typically occurred when two or more alleles were present in the population and represent either intra-strain level genetic heterogeneity or a sequencing artifact. Since there is no definitive way to determine whether these correspond to true allelic heterogeneity or are artifactual, they were discarded without further consideration. Nonetheless, since they are rare, they would have a minimal influence on the outcome of the study.

Single nucleotide variants were further curated by only using trimmed reads that had a mapQ score equal to or exceeding 1.0. The mapQ score is a read quality measurement determined by bowtie during the read mapping step of the pipeline (Langmead and Salzberg, 2012). The mapQ score is also used to validate the consensus base or the most frequent allele at a variable nucleotide site by verifying that it was also the base with the highest average mapQ score across reads exhibiting that base. Finally, a minimum and maximum sequence insert size is used to further curate SNVs. Here, if sequence inserted into the middle of an ORF alignment is too large, the SNVs in that alignment are discarded since the insertion may indicate incorrect alignment of sequences that are separated on the genome. Default parameters of 50 bp and 3× median insert size were used for minimum and maximum sequence insert size, respectively. The total number of SNVs, both inside and outside of ORFs, was determined for each MAG for comparative purposes. However, only SNVs that were within ORFs, as identified by PROKKA, were used for downstream analyses, since these would allow for potential annotation of the encoded proteins. This secondary filtering step was performed to reduce the frequency of false positive SNVs, as the ORFs tended to have much higher read quality than non-coding sequences. Further, SNVs on ORFs are expected to be more informative regarding the functional dynamics of populations and thus more indicative of where selective pressures are acting on the population level genomes.

Curated SNVs among BA1A *Meiothermus* MAGs were collated using the R base package to facilitate downstream comparison. Specifically, a table was uploaded to R that contained (1) each SNV, (2) the nucleotide frequencies of that SNV, (3) the location of that SNV on its ORF, (4) which of the six MAGs contained that SNV, (5) the full nucleotide sequence of the ORF containing that SNV, (6) the inferred amino acid sequence encoded by that ORF, (7) the assigned KEGG ortholog of the inferred protein encoded by that region (where possible), (8) the protein cluster that protein was assigned to, and (9) the KEGG pathways that the protein identified in the preceding step presumably belongs to. This allowed for identification of SNVs shared by multiple MAGs, statistical evaluation of those SNVs, and other relevant analyses within the R statistical platform.

The overall SNV profile was determined for each MAG. Specifically, the total number of SNVs in the MAG was identified, regardless of whether they were on an ORF. Similarly, the number of SNVS in ORFs in those MAGs, the proportion of ORFs in the MAG containing at least one SNV, and the mean and variance of the SNV frequency across the length of the MAG were identified. Student’s T-tests were performed to compare total SNVs among pairs of MAGs, SNVs in ORFs among pairs of MAGs, variance in SNVs per ORF among pairs of MAGs, and the total number of ORFs containing at least one SNV across the designations of community (depth from which it was recovered), abundance (rare versus abundant), and phylogeny (shallow clade versus deep clade, as described below).

### Characterization of SNVs

The six BA1A *Meiothermus* MAGs were compared to identify SNVs that were shared at the same position within an ORF. Shared SNVs identified in all six MAG populations were presumed to be ancestral SNVs that were likely present in the population of founder cells, or those that first colonized the Samail Ophiolite or its geologic precursor, and whose genomes gave rise to the descendent MAGs identified herein. The distribution of SNVs was examined between Type I (S) and Type II (D) MAGs using a hierarchical approach. First, shared SNVs that are present in more than one MAG were identified. This approach was more facile than alignment-based approaches that are more time and computationally intensive. While the latter would identify many more potential SNVs, it would also increase the identification of false positive SNVs. Consequently, SNVs identified using the approaches reported herein should be regarded as a conservative estimate of the total number. Secondly, shared SNVs with the same substitution (i.e., the same consensus base and same variable base) at the same site on the same protein encoding ORF were identified. The number of shared SNVs across these hierarchical categories was subjected to statistical analysis using the R base package. Student’s *t*-tests were used to compare the number of shared SNVs by different MAG pairs or MAG triplets.

### Evaluation of the correlation between shared SNV presence and pairwise MAG sequence similarity

To determine if the presence of shared SNVs corresponded to areas of genomes with high similarity, the ANI of pairs of contigs in pairs of MAGs that contained shared SNVs was determined and compared to the average ANI of pairs of contigs of those same pairs of MAGs that lacked shared SNVs. FastANI (version 1.3.2) (Jain et al., 2018) was used to determine the pairwise ANI of every contig pair between MAGs. Contig pairs containing a shared SNV were then identified. The average ANI for contig pairs containing a shared SNV was then determined for each MAG pair and was compared to the average ANI for contig pairs in that MAG pair that did not contain a shared ANI. The length and coverage of contigs containing shared SNVs and those contigs lacking shared SNVs were also compared to ensure that sequence quality and read depth was not a confounding factor in this analysis. No significant difference was observed between contig coverage (i.e., read depth) between the two groups (*P* = 0.12; two tailed t-test). There was a significant difference in length between contig groups (*P* < 0.01; two tailed t-test), but this is likely a consequence of large sample sizes rather than a true difference, as mean contig lengths were similar (84,325 bp for contigs containing shared SNVs, 50,007 bp for those without), as were the standard deviations (73,698 bp for contigs containing shared SNVs, 63,906 bp for those without). However, since contigs containing less than 80% ANI cannot reliably be aligned, the reported similarity values are intended to be comparative, not an absolute measurement of the actual sequence similarity between specific portions of the genome. Nonetheless, due to the large number of total calculated alignments, the difference in similarity between genomic regions with shared SNVs and those without is expected to be real.

### Estimating the likelihood that shared SNVs arose through convergent evolution

To evaluate the likelihood that SNVs arose in *Meiothermus* MAGs convergently rather than being inherited from a common ancestor (allopatry) or through gene flow/recombination between MAGs, a simple site substitution model was developed and employed. For each MAG pair, the number of SNVs occurring at the same site (i.e., on the same residue on the same ORF) was determined by comparing the retrieved SNVs from InStrain for each MAG. The previously discussed protein clustering was used to identify orthologous proteins and the genes encoding them. This protein clustering approach was utilized in place of a much more computationally intense and error prone whole genome alignment-based approach. The number of shared SNVs that would be expected to have the same base substitution (i.e., the same consensus and variant bases) through convergent evolution (i.e., random mutations and not through horizontal or vertical gene flow) was calculated. Here, roughly 12/256 of SNVs are expected to converge on the same substitution at the same site through random mutation alone. The denominator of 256 (i.e., the 256 total possible pairs of base changes) comes from a base at a given position in the MAG (4 bases are possible) and the possibility of a variant base (4 bases are possible) at this position (4 bases × 4 bases or 16 base combinations). The same applies to the second MAG pair, resulting in 16 combinations × 16 combinations or 256 total combinations for the identity of the original base and what it could mutate to at a single position in both MAGs. The numerator is based on the 16 total pairings where both SNVs start and end with the same base. Thus, the likelihood two SNVs arose from the same substitution is (4 bases choose 2 bases)^2^ or (4)^2^ or 16 minus 4 (the four instances where no base change occurs are subtracted out) is 12. The combined likelihood is therefore 12/256.

For triplet sets of MAGs, the number of possible variations is now based on three mutations rather than two. The likelihood that the three MAGs convergently develop the same SNV at the same position is the combined likelihood that the three MAGs experience a mutation from one nucleotide to another nucleotide (i.e., three MAGs experience the same substitution at the same position in an ORF). Consequently, in the absence of gene flow, it is expected that roughly 12/4,096 of SNVs will converge on the same substitution at the same site. The 4,096 denominator comes from a MAG having an original base at a given position and the possibility of a variant base at this position (4 bases × 4 bases or 16 base combinations). The same is true for the second and third MAGs, resulting in a total of 4,096 combinations (16 × 16 × 16). Thus, the likelihood two SNVs arose from the same substitution is (4 bases choose 2 bases)^2^ or (4)^2^ or 16 minus 4 (the four instances where no base change occurs) or 12. The combined likelihood is therefore 12/4,096.

### Identification of viral and integrated mobile genetic elements (IMGEs)

To identify the quantity and type of viral elements present in the three BA1A subsurface microbial communities, metagenomes were probed for viral nucleotide sequences using VIBRANT (version 1.2.1) (Kieft et al., 2020), which identifies viral sequences by comparing retrieved metagenomic sequences to the KEGG, protein Family (PFAM), and Virus Orthologous Groups (VOG) databases (Thannesberger et al., 2017; El-Gebali et al., 2018; Kanehisa et al., 2020). Contigs from the six BA1A *Meiothermus* MAGs were submitted to the Mobile Genetic Element finder tool (version 1.0.3) (Johansson et al., 2021) to identify the quantity, location, and type of IMGEs present.

## Results

### Overview of previous hydrogeochemical work conducted at BA1A

Hydrological experiments, including flowmeter tests under ambient and forced hydraulic conditions, revealed three discrete depth intervals with connectivity to local aquifers in the 400 m deep BA1A well and these intersected different bedrock types, as reported previously (Lods et al., 2020; Kelemen et al., 2021). Briefly, the 0–30 m depth interval (BA1A30) intersected alluvium (well cased to 21 m to isolate this section) and weathered dunite to at least 30 m, whereas the 41–65 m (BA1A65) and the 108–132 m (BA1A132) depth intervals intersected intact dunite. There was no significant connectivity of upper aquifers to aquifers between 132–400 m depth, as assessed in field with a flowmeter (limit of detection of 0.1 L min^–1^). Field hydraulic tests suggested that waters from the 0–30 m interval may have limited connectivity with those from 41–65 m interval (Lods et al., 2020). Further, it is possible that connectivity exists to the deeper 108–132 m aquifer, although the flow is expected to be substantively less than from the weathered dunite aquifer (0–30 m interval) to the upper (41–65 m) dunite-hosted aquifer (Lods et al., 2020).

The different depth intervals sampled generally corresponded to different geochemical regimes (Lods et al., 2020; Nothaft et al., 2021), with the 0–30 m depth water having a temperature of 34.9°C, a pH of 8.10, a conductivity of 0.458 mS cm^–1^, and an oxidation reduction potential (*Eh*) of 128 mV (Table 1). Similarly, water from the 41–65 m depth interval had a temperature of 35.0°C, a pH of 8.21, a conductivity of 0.402 mS cm^–1^, and an *Eh* of 120 mV. In contrast, water from the 108–132 m depth interval had a temperature of 36.5°C, a pH of 10.67, a conductivity of 0.871 mS cm^–1^, and an *Eh* of −249 mV. Thus, waters from 0–30 m and 41–65 m are classified as Mg–HCO_3_-type waters (Type I) while waters from 108 to 132 m are classified as Ca–OH-type waters (Type II), according to a previously reported classification scheme (Rempfert et al., 2017). Type II waters have extensively reacted with rock (i.e., dunite) in regions of the subsurface thought to be closed to atmospheric inputs whereas Type I waters may include atmospheric inputs (Rempfert et al., 2017).

**TABLE 1:** Select geochemical measurements of waters recovered from discrete depth intervals in well BA1A in February 2019 and their inferred water type.

Isolated depth interval (meters below surface)	Water type	pH	Temperature (°C)	Conductivity (μS cm^–1^)	Redox potential (mV)
0–30	Mg-HCO_3_	8.1	34.9	458	127.7
41–65	Mg-HCO_3_	8.2	35.0	402	120.3
108–132	Ca-OH	10.6	36.4	950	-310.0

Mg-HCO_3_ type fluids are also termed Type I fluids whereas Ca-OH fluids are also termed Type II fluids, based on prior classification schemes (Barnes and O’Neil, 1969; Neal and Stanger, 1985). Values were previously reported in Nothaft et al. (2021).

### BA1A taxonomic composition and diversity

**FIGURE 1:**
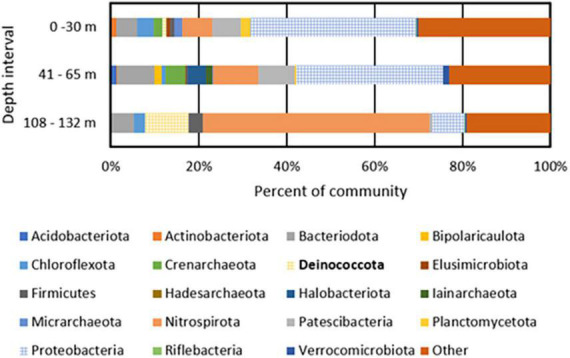
Phylum level taxonomic composition of metagenome assembled genomes (MAGs) recovered from BA1A filtered biomass from discrete depth intervals. MAGs that encode homologs of enzymes or enzyme complexes that allow for the respiration of oxygen (Cox I and II, CydABX) are depicted with a grid pattern. Deinococcota, the phylum containing *Meiothermus*, is bolded in the legend.

A total of 447, 626, and 229 giga base pairs (Gbp) of metagenomic sequence from depth intervals of 0–30 m (BA1A30), 41–65 m (BA1A65), and 108–132 m (BA1A132), respectively, was generated and subjected to assembly and binning (Supplementary Table 1). A similar proportion of each of the BA1A30, BA1A65, and BA1A132 communities was binned (77, 80, and 79%, respectively). In BA1A30, a total of 11 high quality MAGs (i.e., MAGs that had at least 90% completeness and less than 5% contamination) were identified. Thirteen high quality MAGs were recovered from BA1A65, and ten high quality MAGs were recovered from BA1A132. The taxonomic compositions of communities were consistent with those of Nothaft et al. (2021) and showed a decreasing proportion of Proteobacteria with depth and an increasing proportion of Nitrospirae and Deinococcota with depth (Figure 1 and Supplementary Table 2).

The most abundant MAGs in BA1A30 were affiliated with *Parvibaculum* (Proteobacteria; 25% of the binned community), *Burkholderia* (Proteobacteria; 8%), Ignavibacteria (5%), Patescibacteria (3%), and *Thermodesulfovibrio* (3%). The composition of BA1A65 was similar to BA1A30 at the phylum level and was dominated by Proteobacteria, including an unclassified Acidoferrobacterales population (9% of the binned community), *Burkholderia* (7%), and *Lysobacter* (6%). Like BA1A30, Ignavibacteria (5% of the binned community) and *Thermodesulfovibrio* (5%) were also detected in the BA1A65 community. A major shift in taxonomic composition was observed in BA1A132, where *Thermodesulfovibrio* comprised nearly 50% of the binned community. *Meiothermus* was also abundant in BA1A132, comprising roughly 10% of the binned community.

### Inferred oxygen usage among BA1A populations

Metagenome assembled genomes from the discrete depth intervals sampled in BA1A were examined for protein homologs of cytochrome *c* oxidase [Cox I and II; Enzyme Category (EC) 7.1.1.9] and cytochrome *bd* complex (CydABX; EC 7.1.1.7), known protein complexes involved in the respiration of O_2_ (Ludwig, 1987; Jünemann, 1997). MAGs that encoded homologs of one or both complexes were considered as capable of integrating O_2_ into their energy metabolism (i.e., obligate aerobes or facultative anaerobes). MAGs that did not encode homologs of these complexes were considered incapable of integrating O_2_ in their cellular energy metabolism (i.e., obligate anaerobes). Forty-three and 37% of the binned BA1A30 and BA1A65 populations were determined to be aerobic/facultatively anaerobic, respectively, with the remainder inferred to be strict anaerobes (Figure 1). Surprisingly, 20% of the BA1A132 community was determined to be aerobic/facultatively anaerobic based on MAGs encoding Cox; *Meiothermus* comprised 10% of this community. Homologs of proteins that would putatively allow for respiration of additional oxidants (e.g., SO_4_^2–^, S°, Fe(III), NO_3_^–^) were not identified among BA1A *Meiothermus* MAGs (data not shown). In addition to encoding for Cox, BA1A *Meiothermus* MAGs are closely related (83–85% ANI) to the obligately aerobic cultivar, *Meiothermus hypogaeus* (NCBI taxonomy ID: 884155) (Mori et al., 2012), suggesting a similar O_2_-dependent energy metabolism.

To investigate if putative aerobes in BA1A may be capable of generating endogenous O_2_ thereby allowing for a high abundance of putative aerobes in an otherwise anoxic environment, MAGs were probed for genes that encode oxygenic proteins including Nod, Cld, SOD, and Cat (see section “Materials and methods”). Homologs of Cld, including both the pentameric and dimeric forms, were the only such proteins identified in BA1A MAGs. One hundred and fourteen Cld sequences among the BA1A MAGs passed the alignment and conserved residue cutoff used to curate homologs (Supplementary Table 3). All six *Meiothermus* MAGs from BA1A (discussed below) encoded a homolog of Cld, regardless of the depth from which they were recovered.

### *Meiothermus* phylogeny and clade designations

**FIGURE 2:**
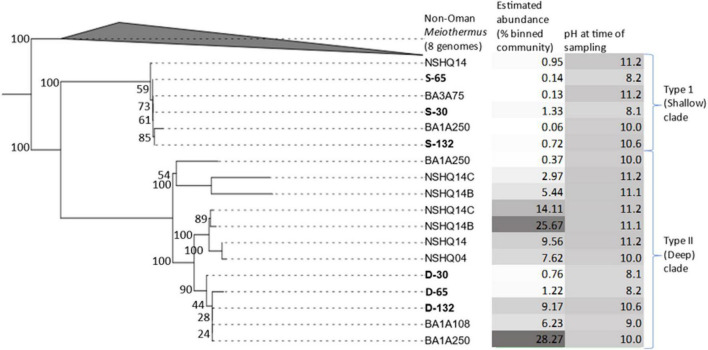
Maximum-likelihood phylogenomic reconstruction of a concatenation of 115 housekeeping genes from metagenome assembled genomes (MAGs) recovered from discrete depth intervals in wells BA1A (sampled with packer system), BA1A (sampled using niskin bottles), BA3A (sampled using niskin bottles), or from specified depths (open well pumping) in NSHQ14B (50 m) and NSHQ14C (85 m). Eight genomes of non-Oman *Meiothermus* served as the outgroup. For BA1A samples collected with the packer system, MAGs are labeled as belonging to the Type I surface phylogenetic clade (S) or the Type II deep phylogenetic clade (D) followed by the maximum depth interval (m) from where they were recovered (30, 65, 132). For BA1A and BA3A samples collected with niskin bottles, MAGs are labeled by well followed by depth of recovery. Only MAGs that were most closely related to *Meiothermus* were considered. MAGs obtained from discrete depth intervals isolated by packers in well BA1A that were used for further genomic analyses are in bold. Bootstrap scores are indicated at each node. The relative abundance of MAGs (% of mapped reads) and the pH of fluids at the time of sampling are indicated, with darker colors corresponding to higher values for both parameters.

Phylogenomic reconstruction of *Meiothermus* MAGs from BA1A, *Meiothermus* from other wells in the Samail Ophiolite study site, and all *Meiothermus* reference genomes available in the Genome taxonomy database (Figure 2) revealed that MAGs from Oman formed a monophyletic clade, with *M. hypogaeus* as the closest non-Oman outgroup. However, *Meiothermus* MAGs from other serpentinizing systems were not yet available to compare phylogenetically to the *Meiothermus* from the Samail Ophiolite. Yet, a single RNA polymerase subunit B (RpoB) sequence from *Meiothermus* was available in the NCBI NR and SRA databases from a metagenome from the Zambales ophiolite (Woycheese et al., 2015). Phylogenetic reconstruction of RpoB homologs from *Meiothermus* MAGs from BA1A, *Meiothermus*-affiliated RpoB from the Zambales ophiolite, and *Meiothermus* RpoB sequences from the NCBI NR and SRA databases revealed the same pattern whereby *Meiothermus* RpoB from the Samail Ophiolite formed a monophyletic lineage, referred to herein as the Oman-specific clade (Supplementary Figure 1).

Within the Oman-specific *Meiothermus* clade, MAGs from BA1A partitioned into two distinct sub-clades. Both *Meiothermus* sub-clades were present in all three depth intervals, but they exhibited opposing abundance trends (Table 2). Specifically, one *Meiothermus* clade, hence referred to as Type II (D) from the BA1A30 (D-30), BA1A65 (D-65), and BA1A132 (D-132) intervals, increased in abundance as depth increased, whereas the other *Meiothermus* clade, hence referred to as the Type I (S) clade comprising BA1A30 (S-30), BA1A65 (S-65), and BA1A132 (S-132), is most abundant in the BA1A30 community and decreased in abundance with depth. Within clade average nucleotide identity (ANI) was 99.99% for each clade, indicating a single population. However, ANI between MAGs forming Type I (S) and Type II (D) clades averaged 84%, whilst the ANI between Oman-specific *Meiothermus* MAGs and *Meiothermus hypogaeus* varied between 78 and 81%, depending on the MAG.

**TABLE 2:** Name, estimated abundance, estimated genome size (mega basepairs, Mbp), estimated number of protein coding genes, strain level metagenomic estimation of growth rate (SMEG), and number of single nucleotide variants detected for metagenome assembled genomes (MAGs) recovered from discrete intervals in well BA1A.

Metagenome assembled genome (MAG)	Estimated abundance (% binned community)	Abundance designation	Estimated genome size (Mbp)	Estimated number of protein coding genes	Inferred genome replication rate (SMEG)	Single nucleotide variants
S-30	1.33	Abundant	3.56	3,278	3.02	1,473
S-65	0.14	Rare	3.25	2,953	2.72	5,016
S-132	0.72	Rare	3.71	3,526	1.00	15,560
D-30	0.76	Rare	2.82	2,656	1.06	6,942
D-65	1.22	Abundant	3.12	2,949	1.04	1,335
D-132	9.17	Abundant	3.00	2,869	1.01	2,589
*M. hypogaeus*	NA	NA	3.70	3,623	NA	NA

MAG names are labeled as belonging to the Type I surface phylogenetic clade (S) or the Type II deep phylogenetic clade (D) followed by the maximum depth interval (m) from where they were recovered (30, 65, 132). MAGs were also empirically defined as abundant versus rare based on read mapping (see section “Materials and methods”). Genome characteristics of the most closely related *Meiothermus* cultivar that is not from Oman, *Meiothermus hypogaeus*, are provided for context. A SMEG score of 1.00 indicates a non-replicating MAG. NA, not available.

### *Meiothermus* inferred genome replication rates

To begin to assess whether *Meiothermus* MAGs detected in each depth interval correspond to cells that are likely active or inactive, genome replication rates were estimated using metagenomic sequence data and SMEG calculations. SMEG scores for five of the six high quality *Meiothermus*-affiliated MAGs in BA1A indicate active genome replication, as indicated by a SMEG score exceeding 1.0 (Table 2). The sole exception was *Meiothermus* S-132, which belongs to the Type I (S) clade but that was recovered from the BA1A132 community. The other two MAGs that belong to the Type I (S) clade, S-30 and S-65, had SMEG values of 3.019 and 2.722, respectively. In contrast, MAGs that belong to the Type II (D) clade that were detected in BA1A30 (D-30), BA1A65 (D-65), and BA1A132 (D-132) communities had SMEG values of 1.060, 1.042, and 1.013, respectively. The 95% confidence intervals for all three Type II (D) clades are above one, indicating that these values are significantly different than 1.000 (non-replicating genome). Thus, while the MAGs corresponding to the Type II (D) clade are in a state of active genome replication, they are doing so more slowly than the Type I (S) clade at the 0–30 m and 41–65 m intervals. Further, the Type I (S) *Meiothermus* MAG S-132, recovered from Type II waters, is not in a state of active genome replication.

### *Meiothermus* genome sizes

To further examine differences in the BA1A MAGs that correspond to the Type I (S) and Type II (D) clades, their genome sizes were inferred. The inferred genome size was similar for MAGs from each respective clade (Table 2). MAGs belonging to the Type I (S) clade had an estimated genome size of 3.5 Mega-basepairs (mbp), which is comparable to the observed genome sizes of most other described *Meiothermus* species, including the closest non-Oman relative, *M. hypogaeus*, which has an observed genome size of 3.7 mbp (Mori et al., 2012). However, the Type II (D) clade MAGs had much smaller inferred genomes, with an average size of 3.0 mbp.

### Protein clustering of BA1A *Meiothermus* MAGs and their inferred metabolism

The larger genome sizes of MAGs that cluster within Type I (S) versus Type II (D) *Meiothermus* clades suggested differences in the functional potential of the associated organisms. To determine the differences in encoded protein content between the six *Meiothermus* MAGs, the inferred proteins encoded in MAGs were subjected to protein clustering and comparative analysis (Supplementary Table 5). The 17,477 inferred protein sequences [encoded by genes or open reading frames (ORFs)] among the six BA1A *Meiothermus* MAGs partitioned into 4,114 clusters, 2,169 of which included protein homologs encoded in both the Type I (S) and the Type II (D) clade MAGs. Among the proteins that were shared between the Type I (S) and the Type II (D) clade MAGs are homologs of proteins for complete glycolytic and tricarboxylic acid cycles as well as homologs of bidirectional NAD^+^/NADP^+^-reducing [NiFe]-hydrogenases. Both clades also encoded a homolog of group 1 [NiFe]-hydrogenases, however, they are likely involved in different processes based on phylogenetic relationships of the large subunit with characterized homologs and the presence/absence of motifs indicative of translocation across the membrane. The Type I (S) clade group 1 [NiFe]-hydrogenase homolog is predicted to be located in the periplasm based on the presence of a twin-arginine translocation (tat) motif and to be a high affinity H_2_ oxidation enzyme (group 1h) that functions in aerobic respiration (Søndergaard et al., 2016). In contrast, the Type II (D) clade group 1 [NiFe]-hydrogenase homolog is predicted to be cytoplasmic or inner membrane-associated (lack of tat motif) and is predicted to be involved in H_2_ oxidation coupled to anaerobic respiration of sulfate, fumarate, nitrate, or metals (Søndergaard et al., 2016). Interestingly, homologs allowing for respiration of such oxidants were not identified among the Type II (D) clade MAGs.

In addition, of the 4,114 protein clusters, 1,196 were unique to the Type I (S) clade MAGs, and 749 were unique to the Type II (D) clade MAGs. Importantly, because these MAGs are incomplete (Supplementary Table 2) it cannot be known confidently that a protein sequence is necessarily unique to a clade. Nonetheless, that they were identified in three Type I (S) MAGs and no Type II (D) MAG and vice versa gives additional confidence to their uniqueness to a given clade. A far greater proportion of the proteins unique to the Type I (S) clade of BA1A *Meiothermus* MAGs were annotated in the KEGG categories of ABC transporters (26× as likely) and amino sugar and nucleotide sugar metabolism (10× as likely). Proteins unique to the Type I (S) clade of *Meiothermus* MAGs were also more likely annotated as two component regulatory systems (10× as likely) or as involved in quorum sensing (4× as likely). A number of protein encoding genes associated with sulfur metabolism were also identified in Type I (S) clade MAGs, including those encoding SoxABCDX that would potentially allow for oxidation of thiosulfate/elemental sulfur. Intriguingly, while these genes are encoded by most non-Oman *Meiothermus* species, including those that are sister to the Oman *Meiothermus* clade (Figure 2), they are absent from the Type II (D) Oman clade. This suggests loss of these genes and the functionalities in the Type II (D) clade.

Proteins unique to the Type II (D) clade MAGs tended to be annotated in the KEGG categories of thiamine biosynthesis (3.5× as likely) and folate biosynthesis (5× as likely). Moreover, a complete thiamine biosynthesis pathway (ThiEMDLGOS and its regulator TenA) was identified exclusively in the Type II (D) clade MAGs. Based on close sequence homology of these subunits to *Acetothermia* and their absence in other non-Oman *Meiothermus* genomes, it is likely that the thiamine biosynthesis pathway was acquired via a horizontal gene transfer event from *Acetothermia* that tend to be enriched in Type II waters (Colman et al., 2022). Additionally, the Type II (D) clade encodes a copy of pyruvate ferredoxin oxidoreductase (PFOR; *porABC*), which, based on sequence homology, may have been obtained via HGT from cohabitating and anaerobic *Thermodesulfovibrio* that often predominates in Type II waters (Templeton et al., 2021). In addition, all six MAGs, regardless of whether they correspond to the shallow or the Type II (D) clade, encode pyruvate dehydrogenase (PDH) that typically facilitates oxidative conversion of pyruvate to acetyl CoA and CO_2_ (unidirectional) in aerobes (de Kok et al., 1998).

### SNV profiles of individual BA1A *Meiothermus* MAGs

To assess the extent of gene exchange among spatially or ecologically differentiated *Meiothermus* populations, a population biology study of MAGs via analyses of SNVs was undertaken. A total of 35,019 curated (see section “Materials and methods”) SNVs were identified among the six BA1A *Meiothermus* MAGs, 22,476 of which were in ORFs (Table 3). SNVs were not distributed evenly across the MAGs. MAGs classified as rare [i.e., are the less abundant *Meiothermus* population in their respective community such as S-132 (Table 2)] had a significantly higher number of ORFs containing SNVs than their abundant counterparts (*P* = 0.00047; two tailed T-test). Further, they had a significantly higher total number of SNVs in ORFs (*P* = 0.012) and a significantly higher mean number of SNV’s per ORF (*P* = 0.0013). There was no significant difference between the mean number of SNVs of MAGs that belonged to different clades [i.e., Type I (S) versus Type II (D)] or that were recovered from the different depths. However, the clade the MAG belonged to [i.e., Type I (S) versus Type II (D)] was a slightly stronger, albeit not significant, predictor of the mean number of SNVs across the MAGs (phylogenetic clade *P* = 0.075; abundance *P* = 0.20).

**TABLE 3:** Single nucleotide variants (SNVs) identified between the specified pair or triplet of metagenome assembled genomes (MAGs).

Metagenome assembled genome (MAG) pair or triplet	Shared SNVs at same site	Shared SNVs at same site with same substitution	Expected number of convergent SNVs^a^	Average ANI between contigs containing shared SNVs	Average ANI between contigs lacking shared SNVs
S-30, D-30	2	0	<1	NA^b^	81.0
S-65, D-65	0	0	0	NA^b^	81.8
S-132, D-132	3	2	<1	95.4	81.5
S-65, S-132	259	219	12.14	100	83.5
D-65, D-132	62	44	2.91	100	83.9
D-30, D-65	25	11	1.17	98.1	82.5
D-30, S-132	73	9	3.42	85.6	81.4
S-30, S-65	25	22	1.17	100	86.6
S-30, D-132	8	0	0.38	NA^b^	81.4
S-30, D-65	37	0	1.73	NA^b^	81.5
S-30, S-132	173	148	8.11	100	84.1
D-65, S-132	34	20	1.59	91.4	82.7
D-30, S-65	15	3	<1	93.8	80.9
D-30, D-132	27	15	1.27	98.4	83.5
S-65, D-132	1	0	<1	NA^b^	81.4
D-30, D-65, D-132 (*deep clade MAGs*)	10	2	<1	NA^c^	NA^c^
S-30, S-65, S-132 (*shallow clade MAGs*)	5	4	<1	NA^c^	NA^c^
S-30, D-65, D-132 (*common MAGs*)	0	0	0	NA^c^	NA^c^
D-30, S-65, S-132 (*rare MAGs*)	1	0	<1	NA^c^	NA^c^

MAGs are labeled as belonging to the Type I surface phylogenetic clade (S) or the Type II deep phylogenetic clade (D) followed by the maximum depth interval (m) from where they were recovered (30, 60, 132). The number of shared SNVs at the same site and those with the same substitution are reported, as is the expected number of SNVs that could arise through evolutionary convergence (see section “Materials and methods”). The average nucleotide identity (ANI) of contigs that share versus those that lack shared SNVs is provided. Bolded pairings or triplets denote MAGs that exhibit evidence for gene flow/recombination.

^a^The number SNVs that would theoretically be present in MAG pairs or triplets due to convergent evolution in the absence of gene flow/recombination as calculated based on substitution frequencies (see section “Materials and methods”) multiplied by the number of SNVs shared by a pair or triplet of MAGs, as identified by InStrain (first column above). Values were rounded to the nearest integer.

^b^ANIs are not reported for pairs of MAGs with no shared SNVs.

^c^ANI calculations were not performed for triplets of MAGs.

#### SNVs shared between MAGs

Of the 4,484 total protein clusters identified among the six *Meiothermus* MAGs, 369 protein clusters had no SNVs within their associated ORF, while 1,322 clusters had at least one SNV in an associated ORF for each *Meiothermus* MAG. The 369 invariable ORFs encode proteins that are highly conserved and/or that are essential (e.g., housekeeping proteins) (data not shown).

There was no significant difference in the number of shared SNVs by MAGs in the same clade or shared by MAGs with similar abundances (Table 2). Pairs of MAGs where both members came from the same depth interval, such as S-30/D-30, S-65/D-65, and S-132/D-132, have significantly fewer shared SNVs at the same site within a protein encoding gene than other pairs. The pairing of S-65/D-65 had no SNVs at the same site whereas the pairing of S-30/D-30 had two SNVs at the same site but with different base substitutions. Finally, the pairing of S-132/D-132 had three SNVs at the same site, two of which had the same base substitution. Surprisingly, two pairs of MAGs (S-65/D-132 and S-30/S-132) have a significantly higher number of shared SNVs in protein encoding ORFs than other pairs of MAGs, with 219 and 148 SNVs at the same site with the same base substitution, respectively (Table 3). This was despite the fact that the organisms with these MAGs have been evolutionarily/spatially more isolated from each other than those organisms from the same aquifer. The limited evidence for shared SNVs in MAGs recovered from the same depth is attributed at least in part to limitations associated with the approach utilized herein, where a given contig is only allowed to be present in a single bin due to the non-redundant nature of the contig binning process. As such, the results presented herein for co-inhabiting populations should be considered a conservative estimate of gene flow/recombination across phylogenetic lineages.

The only triplet sets of BA1A MAGs that had SNVs with the same substitution at the same site within a protein encoding gene occurred among MAGs that belonged to the same phylogenetic clade (Table 3). MAGs comprising the Type II (D) clade had ten SNVs at the same site of a protein encoding gene, two of which had the same base substitution. MAGs comprising the Type I (S) clade had five SNVs at the same site of a protein encoding gene, of which four had the same base substitution.

#### Correlation between shared SNVs and pairwise MAG genome similarity

The pairwise ANI between each contig in pairs of BA1A *Meiothermus* MAGs was calculated to determine if genomic regions containing shared SNVs at the same position were more similar than regions lacking shared SNVs. Importantly, it is also possible to calculate the ANI of individual protein coding genes (i.e., genes encoding proteins among protein clusters) that are shared between MAGs to identify those that likely arose from recent recombination (high ANI compared to average ANI). However, such an approach could confound an assessment of the influence of recombination due to potential differences in selective pressures on encoded proteins (i.e., false-positives among housekeeping genes under purifying selection, false-negatives among protein genes undergoing drift). A comparison of ANIs between contigs with shared SNVs among MAG pairs thus is a more conservative approach to identifying regions of MAGs that have been influenced by recombination and is the approach utilized herein.

**FIGURE 3:**
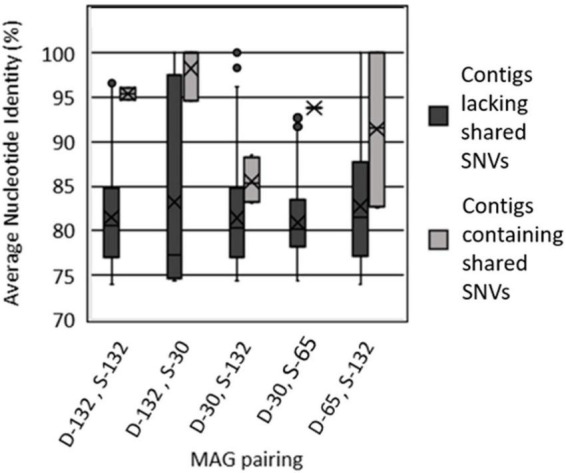
Average nucleotide identity of contigs between select pairs of metagenome assembled genomes (MAGs) that contain shared single nucleotide variants (SNVs) versus contigs from those pairs of MAGs that lack shared SNVs. MAGs are labeled as belonging to the Type I surface phylogenetic clade (S) or the Type II deep phylogenetic clade (D) followed by the maximum depth interval (m) from where they were recovered (30, 65, 132). Values for all MAG pairings are reported in Table 3.

Of the 10 MAG pairings that shared SNVs (Table 3), all exhibited higher genomic similarity in the regions containing those SNVs than in the other regions of the genome (Figure 3 and Table 3). While there is a difference in the average length of contigs containing SNVs, there is no difference in the read depth and overall sequence quality (*P* = 0.12; two tailed T-test). Further, while not every contig pair could be evaluated, as ANI is not reliable for sequences with less than 80% sequence similarity, the sample size of those contigs that were evaluated indicates that while the actual percentage similarities may not be accurate, the overall trend is accurate. In MAG pairs belonging to the same clade, much of this genomic similarity is likely a consequence of shared ancestry. Since the majority of contigs had shared SNVs, their high ANI reflects the overall high ANI between the MAG pairs. However, for the five pairs belonging to different clades but still exhibiting shared SNVs, this increase in genomic similarity near shared SNVs is likely to be a consequence of recombination in at least part of that contig.

#### Likelihood of SNVs arising convergently

A simple site substitution model was developed to evaluate the likelihood that SNVs in MAG pairs or MAG triplets arose convergently as opposed to having been either a property of their ancestor or result from gene flow/recombination (see section “Materials and methods” for model description). Ultimately, the model predicts that 12 out of 256 of all SNVs occurring at the same site between two MAGs and that 12 out of 4,096 of all SNVs occurring at the same site between three MAGs should have the same base substitution. In other words, the expected number of shared SNVs with the same substitution in a pair of MAGs in the absence of gene flow/recombination was calculated as: 12/256 × number of shared SNVs. As an example, the expected number of convergent SNVs between MAG pair D-65 and S-132 that have the same base substitution is ∼2 (12/256 × 34 = 1.59 or ∼2). However, 20 out of 34 shared SNVs at the same site in these two MAGs were found to have the same base substitution (Table 3). This suggests that convergent evolution alone (accounting for only ∼2 of the 20 observed shared SNVs with the same base substitution) is unlikely to explain the prevalence of shared SNVs with the same base substitution at the same site. This is the case for other MAG pairs from different clades (S versus D) (Table 3). This model is an oversimplification of mutation dynamics in natural systems since each base does not have an equal likelihood to mutate to any other base and different organisms and their genomes are not necessarily under the same selective pressures at any given time. Nonetheless, given the number of shared SNV observations, these probabilistic differences should become irrelevant.

Single nucleotide variants shared amongst MAGs belonging to the same clade were also examined (Table 3), as these SNVs were possibly present in the common ancestor of members of the clade, prior to their partitioning across different aquifers with differing chemistry that were intersected by BA1A. Alternatively, it is possible that these also arose from gene flow/recombination. Using the site substitution model (see section “Materials and methods”), the expected number of SNVs exhibiting the same base substitution for pairs of MAGs from the same clade could not be accounted for by convergent evolution alone in any set of MAG pairings, regardless of the clade from which they affiliate (Table 3). That the contigs where shared SNVs with the same base substitution had a higher ANI than regions of the MAGs where shared SNVs were not located could point to gene flow/recombination between these populations as well. However, given that the majority of contigs among MAG pairs contained shared SNVs, it is equally possible that these were inherited from their ancestral population.

The site substitution model was also applied to triplet MAGs from either the same clade or from different clades. In the case of the triplet MAGs from the Type I (S) clade, the expected number of SNVs exhibiting the same base substitution that arose convergently was estimated at < 1 (12/4,096 × 5 shared SNVs = 0.0146 or <1; Table 3). Similarly, the expected number of SNVs exhibiting the same nucleotide for triplet MAGs from the Type II (D) MAGs that arose convergently is <1 (12/4,096 × 10 shared SNVs = 0.029). These expected values are both less than the number of observed shared SNVs with the same base substitution [4 and 2 for Type I (S) and Type II (D) clades; Table 3] indicating that they are likely the result of gene flow/recombination or common ancestry rather than convergent evolution.

This same model was applied to each other MAG pair and MAG triplet to determine the likelihood that a SNV arose convergently, rather than being a result of gene flow/recombination or common ancestry. The number of expected convergent SNVs varied from near 0 (S-65/D-65 pairing) to 12 (S-65/D-132 pairing; Table 3). Ultimately, nine out of the total 15 possible pairs of *Meiothermus* MAGs exhibited probabilistic evidence for gene flow/recombination or shared ancestry as evidenced by observed shared SNVs with the same substitution exceeding expected values based on convergence alone. Seven of these nine pairs were from different depths and, more importantly, from different clades.

### Identification of viral and non-viral integrated mobile genetic elements (IMGEs) in BA1A communities

Metagenome assembled genomes were evaluated for virus signatures and IMGEs. Within the BA1A30, BA1A65, and BA1A132 communities, 4,610, 7,969, and 4,482 viral sequences, respectively, were identified. However, none of the retrieved viral sequences were found on *Meiothermus* contigs. Nonetheless, 19 total IMGEs were identified on *Meiothermus* contigs, 13 of which were classified as insertion sequences (i.e., a transposase gene flanked by two inverted repeats) while the remaining six were classified as composite transposons or a composite mobile genetic element resulting from a transposase acting on the inverted repeat of a related mobile genetic element (MGE) that transposes both the original and new element together (Clark et al., 2019). *Meiothermus* MAGs from BA1A that comprised the Type II (D) clade consistently had more IMGEs than those that comprised the Type I (S) clade. Of the 19 IMGEs, four were present in more than one MAG from the Type II (D) clade. The first shared IMGE, ISGlo6, is present in D-132, D-65, and S-132. The sequence includes the transposase InsK, which may be able to act on additional insertion sequences besides ISGlo6 (Fernández De Henestrosa et al., 2000). The D-132 and D-65 sequences exhibited 100% identity (Table 4), but the S-132 MAG sequence is truncated and comprised only 131 residues compared to the full 214 residues in the other MAGs. Further, the S-132 MAG ISGlo6 sequence shared only 61% sequence identities with that of D-132 and D-65 MAGs. In the D-132 and D-65 MAGs, the insertion sequence is preceded by upwards of 10 kilo basepairs (kbp) that exhibited 100% sequence identity, which may constitute part of a larger transferred sequence.

**TABLE 4:** Percent shared nucleic acid sequence identities between pairs of mobile genetic elements (MGE) retrieved from BA1A *Meiothermus* metagenome assembled genomes (MAGs).

	IS640	ISGlo6	ISMyca1	ISPa85	ISPlu21	ISUNCu3
D-132	NA	1.00	1.00	NA	1.00	NA
D-65	NA	1.00	1.00	NA	NA	NA
D-30	1.00	NA	0.83	1.00	1.00	NA
S-132	NA	0.61	0.42	NA	NA	1.00
S-65	NA	NA	NA	NA	NA	NA
S-30	NA	NA	NA	NA	NA	NA

MAGs are labeled as belonging to the Type I surface phylogenetic clade (S) or the Type II deep phylogenetic clade (D) followed by the maximum depth interval (m) from where they were recovered (30, 65, 132). The MAGs encoding MGEs are in rows and the individual MGEs are in columns. The percent nucleic acid identity of the specified MGE in each MAG is given in reference to the MGE from MAG D-132, or from whichever MAG encoded the MGE when it was absent from D-132. NA, not available.

The second shared IMGE, ISMyca1, was present in D-30, D-65, D-132, and S-132 MAGs (Table 4). The D-132 and D-65 MAG ISMyca1 sequences are identical. Moreover, the shorter D-132 MAG contig containing the ISMyca1 exhibited 100% sequence similarity to a portion of the larger D-65 MAG contig. The D-30 MAG ISMyca1 sequence, however, shared only 83% sequence identity with the D-132 and D-65 MAG ISMyca1 sequences. The S-132 MAG ISMyca1 sequence exhibited less than 50% similarity to the other three ISMyca1 sequences and lacked an identifiable transposase. The third potentially shared IMGE is ISPlu21 (Table 4), which is present in the D-30 MAG and that was duplicated in the D-132 MAG, one on the sense strand and one on the antisense strand. All three ISPlu21 sequences exhibited 100% sequence identity and are surrounded by upwards of 10 kbp also bearing 100% sequence identity.

The fourth sequence is Tn125, a common composite mobile genetic element with a wide phylogenetic distribution (Acman et al., 2022). One copy of Tn125 was detected in each of the six BA1A *Meiothermus* MAGs. Intriguingly, the degree of similarity between these Tn125 sequences does not recapitulate the presumed evolutionary history of the six BA1A *Meiothermus* MAGs, based on housekeeping genes or RpoB proteins (Table 5 and Figure 2). The Tn125 sequences of the D-132 and D-65 MAGs are identical but exhibited only 43% sequence identity to the Tn125 sequence present in the D-30 MAG. Further, the D-132 and D-65 MAG Tn125 sequences exhibited 87% identity to the S-65 MAG Tn125 sequence. Similarly, S-132 and S-30 MAG Tn125 sequences exhibited 100% identity to each other, 44% identity to the S-65 MAG Tn125 sequence, and 87% identity to the D-30 MAG Tn125 sequence.

**TABLE 5:** Percent nucleic acid identities between pairs of transposon 125 (Tn125) sequence homologs retrieved from BA1A *Meiothermus* metagenome assembled genomes (MAGs).

	D-132	D-65	D-30	S-132	S-65	S-30
D-132	1.00	1.00	0.42	0.44	0.86	0.43
D-65	–	1.00	0.41	0.43	0.87	0.42
D-30	–	–	1.00	0.88	0.44	0.87
S-132	–	–	–	1.00	0.43	1.00
S-65	–	–	–	–	1.00	0.44
S-30	–	–	–	–	–	1.00

MAGs are labeled as belonging to the Type I surface phylogenetic clade (S) or the Type II deep phylogenetic clade (D) followed by the maximum depth interval (m) from where they were recovered (30, 65, 132).

## Discussion

Spatially segregated microbial communities from three depth intervals spanning 132 m in BA1A were shown to contain abundant populations of *Meiothermus*, a genus that thus far comprises only obligately aerobic cultivars (Mori et al., 2012). This was true even in the highly reduced Type II waters encountered at depth in BA1A (>132 m). While perhaps surprising, these observations align with previous 16S rRNA gene (Rempfert et al., 2017; Nothaft et al., 2021) and metagenomic sequencing studies (Fones et al., 2019, 2021; Kraus et al., 2021) that also revealed the presence of *Meiothermus* in anoxic Type II waters in the Samail Ophiolite. For example, sequences affiliated with *Meiothermus* were detected in highly reduced fluids from samples collected from depths of 85 m in well NSHQ14 where Type II waters are encountered (Rempfert et al., 2017; Fones et al., 2021). Like the Samail Ophiolite, investigations of highly reduced fluids from the Cedars, Zambales Ophiolite, and Lobios hot springs, all of which are influenced by serpentinization, also identified *Meiothermus* sequences (López-López et al., 2015; Woycheese et al., 2015; Suzuki et al., 2017).

Several non-mutually exclusive explanations were put forth to account for the presence of *Meiothermus* in highly reduced waters including (1) *Meiothermus* was present in Type II waters due to inadvertent mixing with Type I waters during their collection and thereby represent inactive cells in said waters, (2) distinct populations of *Meiothermus* exist in Type I and Type II waters in the Samail Ophiolite that are indistinguishable via the sequence of the conservative 16S rRNA marker gene, (3) *Meiothermus* in Type II waters are aerobes capable of generating endogenous O_2_ to fuel their energy metabolism, (4) *Meiothermus* in Type II waters are facultative anaerobes, and/or (5) that there is more connectivity among the 0-30 m, 41-65 m, and 108-132 m aquifers than expected based on geochemical and hydrological data (Lods et al., 2020; Nothaft et al., 2021). More connectivity of aquifers (explanation 5) would potentially allow for movement/dispersal of *Meiothermus* cells, viruses, or DNA between surface and subsurface aquifers that could impact the extent of gene flow/recombination and shape patterns of speciation.

The use of packers to collect samples from discrete intervals in BA1A, combined with SMEG scores indicating that abundant Type II (D) MAGs are in an active state of genome replication at depth (albeit at low levels), discounts explanation 1 as a source of abundant *Meiothermus* in the 108-132 m depth interval community in BA1A (Nothaft et al., 2021). Importantly, the detection of Type I (S) *Meiothermus* (i.e., S-132), which was not in an active state of genome replication in the 108-132 m depth interval at the time of sample collection, could be due to residual water from mixing during drilling, small ambient downflow after drilling, or sources of vertical flow (other than from the borehole) from shallower aquifer levels during pumping with packers. This possibility is suggested based on a geochemical model that indicated that ∼7.0% of the 108-132 m fluids are of the Type I, Mg-HCO_3_ end member type (Nothaft et al., 2021). Regardless, such possibilities cannot explain the increased prevalence of Type II (D) *Meiothermus* with depth in BA1A.

**FIGURE 4:**
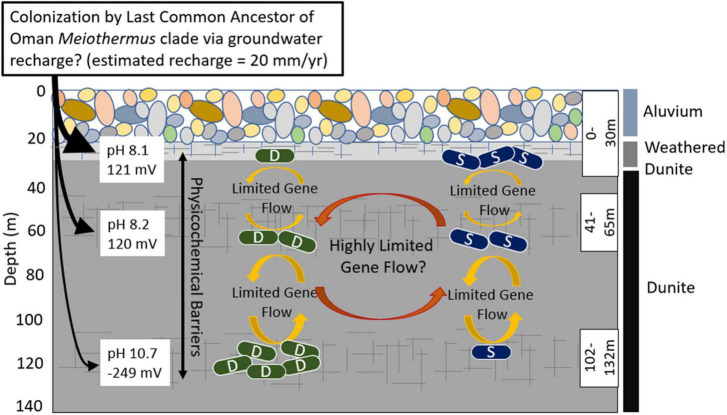
Schematic depicting proposed evolutionary dynamics of the Type I surface (S) populations and the Type II deep (D) populations of *Meiothermus* in three discrete depth intervals (fractures, indicated by hashed lines, isolated using a down borehole packer system) in well BA1A of the Samail Ophiolite and potential gene exchange/recombination between them and selective pressures indicated by arrows and text. The number of S and D cells at each depth interval is meant to depict the relative abundance of populations belonging to each clade at that depth. Characteristics of the waters and the predominant rock types that they interact with are indicated. The evidence for highly restricted gene flow/recombination (limited number of shared SVNs) between S and D *Meiothermus* populations from the same depth interval could be an artifact of the binning process, as discussed in the text, and the reader is cautioned of this possibility through the use of the question mark following this statement. Single cell genomics, in combination with other physiological approaches, would provide a more robust mechanism to evaluate whether the evidence for limited gene flow/recombination is artifactual or is due to selection against hybrid genotypes.

Phylogenetic analyses of *Meiothermus* from the three depth intervals in BA1A, other previously sampled fractured fluids from the Samail Ophiolite (Fones et al., 2019, 2021; Kraus et al., 2021), and other serpentinite systems was performed to begin to evaluate explanation 2. Phylogenomic analysis of housekeeping genes recovered from Oman MAGs revealed a monophyletic clade comprising two sublineages, to the exclusion of *Meiothermus* from other environments. This was also found to be true when *Meiothermus* RpoB sequences from BA1A, additional wells in the Samail Ophiolite, and a single other serpentinite (Zambales Ophiolite) were examined. Unfortunately, attempts to do a similar analysis of 16S rRNA genes recovered from BA1A MAGs, from MAGs from other Oman wells, and those in the NCBI NR and SRA database were unsuccessful due to the highly fragmented nature of those genes in metagenome assemblies (data not shown). This indicates that distinct *Meiothermus* phylotypes exist in the Samail Ophiolite and that these were not distinguished in previous studies via analyses of the more conservative 16S rRNA marker gene. Together, these observations suggest that an ancestral *Meiothermus* population colonized the Samail Ophiolite or its geologic predecessor (Figure 4) and likely underwent speciation that led to two sublineages that have only been identified in the Samail Ophiolite to date. Additional metagenomic sequencing of other globally distributed ophiolites will be required to determine if these two sublineages are truly endemic to the Samail Ophiolite. These two sublineages of MAGs were termed Type I (S) and Type II (D) clades to reflect the water type (Type I versus Type II) and depth [Shallow (S) versus deep (D)] where they were most abundant (Figure 4).

To begin to investigate explanations (3 and 4) above, MAGs were compared and subjected to metabolic reconstruction, with the specific aim of determining if MAGs were capable of aerobic or anaerobic metabolism and/or generating their own endogenous O_2_. Type II (D) MAGs are ∼14% smaller than the Type I (S) MAGs, a finding that is potentially consistent with genome streamlining to reduce the nutrient and energetic costs associated with genome replication. Genome streamlining would presumably be advantageous in the carbon and oxidant limited Type II waters, such as has been suggested previously for populations inhabiting such waters in the Samail Ophiolite (Fones et al., 2019, 2021; Colman et al., 2022). Despite differences in genome sizes, MAGs from both *Meiothermus* clades encode Cox homologs, indicating an ability to respire O_2_. Intriguingly, members of both clades also encode homologs of chlorite dismutase that, in other organisms, have been shown to function in the generation of endogenous O_2_ via dismutation of chlorite (Bardiya and Bae, 2011; Mlynek et al., 2011; Hofbauer et al., 2014; Celis et al., 2015). Such a mechanism could conceivably be used to power aerobic metabolism in the presence of sufficient chlorine oxyanions. However, attempts to measure perchlorate and chlorite in Oman fracture waters have been unsuccessful to date (Templeton, unpublished data), suggesting they are either maintained at undetectable levels or that these compounds are not present in the system. Further, attempts to cultivate *Meiothermus ruber* (ATCC strain 35948), which also encodes Cld, and to enrich *Meiothermus* from Oman fracture fluids under anaerobic conditions with a variety of electron donors/carbon sources and chlorite, chlorate, and perchlorate have been unsuccessful to date (Munro-Ehrlich, unpublished data). Together, these observations point to endogenous production of O_2_ by *Meiothermus* (explanation 3) as being an unlikely reason for the abundance of *Meiothermus* sequence in BA1A132 communities.

The inferred proteomes indicate that the Type I (S) and Type II (D) *Meiothermus* are capable of aerobic, heterotrophic growth. However, the Type II (D) *Meiothermus* MAGs intriguingly encode several proteins suggestive of an ability to potentially grow anaerobically and/or autotrophically. This includes a group 1 [NiFe]-hydrogenase homolog that is predicted to provide reducing equivalents for the anaerobic respiration of sulfate, fumarate, nitrate, or metals (Søndergaard et al., 2016). Further, unlike Type I (S) MAGs, Type II (D) MAGs encode PFOR that allows for the anaerobic conversion of pyruvate to acetyl CoA and CO_2_ (Furdui and Ragsdale, 2000; Fuchs, 2011; Mall et al., 2018; Witt et al., 2019). PFOR is a thiamine-dependent iron-sulfur ([Fe-S]) cluster-containing enzyme (Pieulle et al., 1995) which may, at least in part, explain why Type II (D) MAGs also encode a complete thiamine biosynthesis pathway, unlike Type I (S) MAGs.

Pyruvate ferredoxin oxidoreductase is reversible, allowing for the conversion of acetyl CoA and CO_2_ to pyruvate (Furdui and Ragsdale, 2000; Fuchs, 2011; Mall et al., 2018; Witt et al., 2019). When operating in this direction, it is often termed pyruvate synthase and this enzyme/activity is required for three of the six known autotrophic pathways of CO_2_ fixation including (i) the reductive acetyl CoA pathway [Wood-Ljungdahl pathway (Furdui and Ragsdale, 2000; Fuchs, 2011)], (ii) the reverse tricarboxylic acid (rTCA) cycle (Fuchs, 2011) and the more recently discovered functional reversal of the oxidative TCA cycle, termed the reverse oxidative TCA cycle or roTCA (Mall et al., 2018; Nunoura et al., 2018), and (iii) the dicarboxylate/hydroxybutyrate (DC/HB) cycle (Fuchs, 2011). In the anaerobe *Desulfurella acetivorans* when growing via the roTCA cycle, the directionality of PFOR is dictated by the availability of organic carbon. When acetate is present, heterotrophic growth (pyruvate oxidation) is favored whereas autotrophic growth is favored in its absence (Mall et al., 2018; Nunoura et al., 2018). The distribution and abundance of Type II (D) *Meiothermus* MAGs in anoxic waters in BA1A and in other waters impacted by serpentinization (discussed above), when combined with these MAGs uniquely encoding PFOR, oxidative [NiFe]-hydrogenase enzyme homologs that are typically involved in anaerobic respiration, and full TCA cycles that could potentially be reversed (e.g., roTCA) points to the intriguing possibility that these organisms are capable of anaerobic growth and/or autotrophy. Additional physiological and/or cultivation experiments are needed to further evaluate the plausibility that Type II (D) *Meiothermus* MAGs are facultative anaerobes (explanation 4) and possibly capable of autotrophic growth.

The recovery of Type II (D) MAGs in near-surface, less reacted aquifer waters (0–30 m, 41–65 m) and Type I (S) MAGs in deep, more reacted aquifer waters (108–132 m) suggested the possibility of dispersal between aquifers (explanation 5) and prompted a population-level analysis to examine the extent of gene flow/recombination among MAGs. Surprisingly, few shared SNVs with the same base substitution were identified among pairs of MAGs from different clades that co-occurred in the same depth. Interestingly, shared SNVs with the same base substitution were more prevalent among MAGs from different clades from differing depths. These shared SNVs could not be accounted for based on convergent (random) evolutionary processes alone indicating a potential role for limited gene flow/recombination among depth stratified populations despite chemical and physical barriers to dispersal (Figure 4). The role of gene flow/recombination, as opposed to shared ancestry, is potentially bolstered by the observation that contigs from MAG pairs where shared SNVs are located had, on average, significantly higher ANI than those contigs that did not share SNVs. While signatures of viruses were not evident in BA1A MAGs, several classes of IMGEs including the insertion sequences ISplu6 and the composite transposon Tn125 were detected thereby providing a potential mechanistic explanation for the observed but limited gene flow/recombination between *Meiothermus* populations comprising different clades. Intriguingly, the pattern of sequence identities among IMGEs, including that of Tn125, do not recapitulate the evolutionary history of the *Meiothermus* MAGs from which they derive. This provides further evidence for relatively recent gene flow/recombination between depth-stratified *Meiothermus* populations belonging to the same clade and, to a lesser extent, members of the different clades among depth intervals. The time scales over which gene flow/recombination occurred among spatially segregated *Meiothermus* is expected to be much shorter than the estimated residence times of Type II waters in the Samail Ophiolite [>20,000 years (Paukert Vankeuren et al., 2019)] based on the amount of time since they were in contact with the atmosphere. Such differences are likely a consequence of geochemical measurements made on bulk fluids as opposed to the resolution provided by the sensitive genomic techniques utilized herein.

Taken together, these findings suggest chemical and physical gradients in the subsurface of the Samail Ophiolite (or a geologically similar precursor) facilitated parapatric speciation of *Meiothermus* (Figure 4), a process where co-inhabiting populations speciate to inhabit specific ecological niches, with limited gene exchange between them (Butlin et al., 2008). In the case of *Meiothermus* in Oman, it is suggested that chemical variation generated by serpentinization creates opportunities for spatial variation between populations to develop (a form of geographic isolation), and this spatial variation of populations represents the starting point for further genetic divergence and speciation. As serpentinization of host rock progresses, the chemistry of deep fluids and near surface fluids further diverge, allowing for and/or promoting additional genetic divergence. Concomitantly, the porosity of host rocks (e.g., dunite, harzburgite), which likely become even less porous as serpentinization reactions progress (Klein and Le Roux, 2020), likely limits further dispersal and gene exchange. In this scenario, spatially and ecologically fragmented *Meiothermus* populations adapted to local environmental conditions and thereby independently accumulated distinct mutations, single nucleotide variants, and as illustrated above, differences in encoded functionalities. Parapatric speciation is thus likely to be a prominent mode of evolution in the Samail Ophiolite and other active subsurface geologic systems that have limited spatial connectivity and whose physical and chemical features are in actively evolving or changing.

## Data availability statement

The datasets presented in this study can be found in online repositories. The names of the repository/repositories and accession number(s) can be found in the article/Supplementary material.

## Author contributions

DN and EB collected the samples. AT, JM, and EB designed the experiment. EF extracted DNA. MM-E conducted all bioinformatics analyses. EB and MM-E wrote the manuscript with help from other co-authors. All authors contributed to the article and approved the submitted version.
