# Supplementary material for: Parapatric speciation of Meiothermus in serpentinite-hosted aquifers in Oman
Source: Front Microbiol. 2023 Apr 12;14:1138656. doi: 10.3389/fmicb.2023.1138656 (PMC10130571; doi:10.3389/fmicb.2023.1138656)
Supplement: Supplementary file 1 [file Data_Sheet_1.DOCX]

**Supplementary Materials and Methods**

**Site Description and Drilling.** The 0.152 m diameter, 400 m deep rotary-drilled well, BA1A, in the Samail Ophiolite, Sultanate of Oman, was drilled between February 20^th^ and March 2^nd^, 2017. BA1A is a part of a multi-borehole observatory established by the Oman Drilling Project. Details on drilling are reported previously (Lods et al., 2020; Kelemen et al., 2021). Briefly, BA1A was cased to 21 meters to isolate alluvium near the surface and the well intersects the mantle section of the ophiolite at depth. The well intersects fully serpentinized dunite in the upper 250 m and partially serpentinized harzburgite at greater depth (Lods et al., 2020; Kelemen et al., 2021). The well was subject to air lifting immediately after drilling was completed in attempt to clean the well of drilling fluids. This was done prior to hydrological, chemical, and microbial characterization of isolated fracture waters obtained using an inflatable packer system in 2018 and 2019 (samples analyzed herein) (Lods et al., 2020; Nothaft et al., 2021), as described below.

**Sampling and Field Measurements.** A Solexperts packer system (Zurich, Switzerland) that included two inflatable bladders (“packers”) and a Grundfos (Bjerringbro, Denmark) model SQE 1–140 submersible pump was installed in BA1A in February 2019, enabling the isolation of discrete depth intervals for hydrological testing (Lods et al., 2020) and microbiological and chemical characterization of groundwaters (Nothaft et al., 2021). A volume of water in subsurface pipes or more was pumped and discarded prior to collecting samples of groundwater. Groundwaters were subjected to measurements of temperature, pH, electrical conductivity, and oxidation-reduction potential (*Eh*) during sampling. These data are reported previously (Nothaft et al., 2021) and are presented herein (**Table 1**) to provide context.

Samples of planktonic biomass were collected from discrete depth intervals in February 2019 using the packer system, as described previously (Nothaft et al., 2021). Briefly, biomass was concentrated from 5 to 20 L of groundwater by pumping through sterile 47 mm diameter, 0.22 µm pore size Millipore (Burlington, MA) polycarbonate inline filters. Filters containing biomass were placed in sterile cryovials using flame-sterilized forceps and these were placed in liquid N_2_ dewars for transport back to the lab. At the lab, cryovials and their contents were stored at -80°C until the filters were subjected to DNA extraction.

**DNA Extraction and Shotgun Metagenomic Sequencing**. Genomic DNA was extracted from filtered biomass with the Qiagen PowerSoil kit (Germantown, MD) following manufacturer instructions, with the exception that a 30s bead beating step using a FastPrep®-24 (MP Biomedicals, Santa Ana, CA) homogenizer (instead of a standard vortexer) was added to improve cell lysis. Negative control extractions of filters were conducted. Genomic DNA was quantified via the high sensitivity Qubit assay (Thermo Fisher Scientific, Waltham, MA) and no detectable DNA was measured in negative control extractions. DNA was submitted to the University of Wisconsin Biotechnology Center for library preparation following the Illumina (San Diego, CA) regular fragment (~300 bp) kit and these libraries were shotgun sequenced via the Illumina NovaSeq 6000 (2 x 150 bp) platform. Information on the depth and quality of sequences obtained from the three libraries are reported in **Table S1**.

**Metagenomic Assembly and Binning.** Raw sequence reads were trimmed using TrimGalore (version 0.6.5) (https://www.bioinformatics.babraham.ac.uk/projects/trim_galore/) and were screened for quality using bbmap (version 38.96) specifying a target read depth of 100 and a minimum read depth of 5 to reduce read redundancy. Trimmed reads were then assembled with Spades (version 3.15.4) specifying default parameters with the exception of the -meta option which optimizes the assembly for metagenomic sequence data (Bankevich et al., 2012). Assembled contigs were indexed and subjected to read mapping using bowtie2 (version 2.4.5) to determine contig read depth (Langmead and Salzberg, 2012). Binning of contigs into metagenome assembled genomes (MAGs), including determination of MAG abundance via mapping of curated (non-assembled) reads, was performed using Metawrap (version 1.3) (Uritskiy et al., 2018). Protein calling was performed using PROKKA (version 1.14.5) to identify open reading frames (ORFs) in MAGs and to assign first order annotations to the proteins encoded by those ORFs (Seemann, 2014). MAGs are available from National Center for Biotechnology Information (NCBI) under the BioProject identification number PRJNA918706.

**MAG Metabolic Predictions.** MAGs were characterized as belonging to putatively aerobic or anaerobic cells via several complementary approaches. The potential for BA1A populations to integrate O_2_ into their energy metabolism (aerobes, facultative anaerobes) was first assessed by examining MAGs for homologs of cytochrome *c* oxidase (Cox I and II; Enzyme Category (EC) 7.1.1.9) via the protein Basic Local Alignment Search Tool (BLASTp). The cytochrome *c* oxidase sequences from the conserved NCBI protein cluster PCLA_865600 were used as query sequences. Cox is used to respire O_2_ in most aerobic or facultatively anaerobic bacteria and archaea (Ludwig, 1987). MAGs were also examined for homologs of the cytochrome *bd* complex (CydABX; EC 7.1.1.7) using sequences from the NCBI protein cluster PCLA_2846118 as query sequences. In many organisms, CydABX is used to reduce O_2_ as a detoxification strategy (Jünemann, 1997) while in other organisms (currently only described in Bacteria) it can be used to respire O_2_ (Borisov et al., 2011). Thus, MAGs that encoded CydABX were conservatively labeled as aerotolerant unless physiological or biochemical evidence indicated that this complex in a closely related taxon is used to respire O_2_.

Proteins encoded by MAGs were also uploaded to the Kyoto Encyclopedia of Genes and Genomes (KEGG) server for annotation and pathway prediction. Specifically, orthologs predicted by KEGG to be involved in other (i.e., those not involving O_2_) aspects of putative electron transport chains were examined, in particular orthologs of terminal oxidases (e.g., dissimilatory nitrate reductase, dissimilatory bisulfite reductase). The potential for a MAG to correspond to an aerobe or an anaerobe was then cross checked against the metabolism of its closest cultivated relative, as assessed using the Genome Taxonomy Database-Toolkit (GTDB-Tk). Some MAGs were too distantly related (i.e., bearing less than 80% average amino acid identity (AAI) to a housekeeping gene (e.g., beta subunit of RNA polymerase (RpoB)) to entries in the GTDB to perform this cross-validation and in such cases, the assignments relied solely on BLASTp and KEGG-based analyses. The taxonomic affiliations, relative abundances, and completeness/quality of MAGs in each of the three communities are reported in **Table S2**.

**Identification and Verification of Alternative Oxygen Production Strategies.** The abundance of putative aerobes in highly reduced subsurface waters of the Samail Ophiolite prompted examination of alternative O_2_-producing biochemical mechanisms in BA1A populations. MAGs were examined for genes encoding enzymes known to produce O_2_, including nitric oxide dismutase (Nod; (Zhu et al., 2019)), superoxide dismutase (SOD; (Imlay, 2002)), chlorite dismutase (Cld; (Hofbauer et al., 2014)), and peroxidases/catalases (Cat; (Singh et al., 2008)) using BLASTp, with characterized proteins as query sequences **(Table S3)**. The distribution of photosystem II reaction center proteins was not investigated as these communities are from the subsurface and are not exposed to photosynthetically active radiation. Retrieved Cld sequences were aligned using MEGA (version 11.0.11) (Kumar et al., 2018) against Cld query sequences that have been empirically demonstrated to dismutate chlorite (Hofbauer et al., 2014; Celis et al., 2015), and only homologs with an alignment score of >250 were retained. Cld homologs were inspected for an Arg173 residue, which is essential for protein function (Mlynek et al., 2011). Cld homologs lacking the essential Arg173 residue were discarded without further consideration. Since no homologs of Nod were identified, additional alignment-based verification steps were not conducted. While many homologs of SOD and Cat were identified, none of the homologs had significant sequence similarity to cytochrome *c* peroxidase (Ccp). Thus far, Ccp is the only dismutase, peroxidase, or catalase shown to link to the quinone pool and facilitate respiration of superoxide (Khademian and Imlay, 2017). As such, SOD and Cat homologs that were identified were presumed to act in a detoxification role rather than a respiratory role.

**Clustering of *Meiothermus* Proteins.** Protein clustering was used to identify protein encoding genes unique to each *Meiothermus* MAG and to facilitate downstream evaluation of SNV profiles. All called proteins for each MAG were subjected to protein clustering using CD-HIT (version 4.8.1) (Fu et al., 2012) resulting in clusters of homologous proteins. Clustering was initially performed using 80% sequence similarity to identify clusters of orthologous proteins (Zaslavsky et al., 2016). After this initial clustering, a second round of clustering was performed using a 40% sequence similarity cut-off to capture paralogous proteins clusters. The validity of assigned clusters was manually verified for randomly selected clusters by evaluating if all proteins in the same cluster shared similar annotations as designated by PROKKA (version 1.14.5). Validity was further assessed by randomly selecting clusters and submitting each protein in the cluster to a pairwise BLASTp analysis. If the results of the pairwise BLASTp had output scores of greater than 10e^-30^, wherein the database for the e-value determination was the set of all proteins in the three metagenomes, that cluster was determined to be valid. This cutoff was chosen because this corresponds to the empiric cluster cut-off used to by NCBI to assign homologous protein clusters (Klimke et al., 2009).

**Characterization of *Meiothermus* Protein Clusters.** Representative sequences from each protein cluster among BA1A *Meiothermus* MAGs were uploaded to KEGG and analyzed using the BlastKOALA annotation pipeline (Kanehisa et al., 2020). This pipeline assigns hierarchical annotations to protein sequences, wherein each uploaded protein sequence is annotated with the closest database protein, and then is placed into a hierarchy of biological pathways. These annotations were collated and compared across the six *Meiothermus* MAGs from BA1A to identify which KEGG orthologs, protein families, and biological pathways were overrepresented in certain MAGs or MAG groupings. Differences between the Type I (S) and Type II (D) clades were investigated, as were differences between more abundant and less abundant (i.e., rare) MAGs.

**Supplemental Figures**


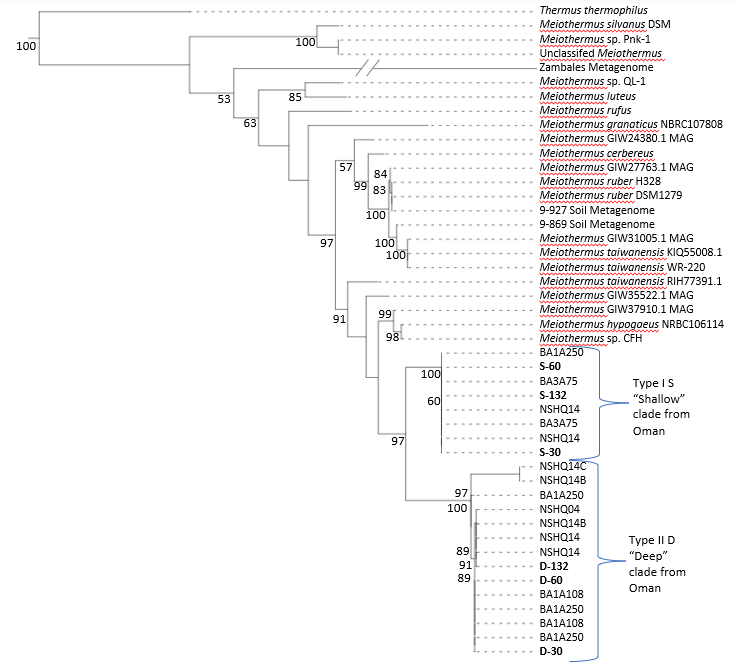


**Figure S1.** Maximum-likelihood phylogenomic reconstruction of RpoB amino acid sequences from metagenome assembled genomes (MAGs) recovered from discrete depth intervals (fractures isolated by packers) in wells BA1A and BA3A (depth follows name, sampled using niskin bottles) from specified depths (open well pumping) in NSHQ14B (50 m) and NSHQ14C (85 m), from *Meiothermus* cultivars, or from assembled metagenomes from non-Oman environments. Only MAGs that were most closely related to *Meiothermus* were considered. MAGs obtained from discrete depth intervals isolated by packers in well BA1A that were used for further genomic analyses are in bold. RpoB from *Thermus thermophilus*, a member of the sister genus *Thermus*, is provided as an outgroup. 1000 Bootstraps were performed, and bootstrap values exceeding 50% are indicated. A RpoB sequence from the Zambales ophiolite is included. A hash was introduced to account for the unusually long branch length of the sequence, possibly due to a sequence artifact.
